# Impact of Vitamin E Supplementation on High-Density Lipoprotein in Patients With Haptoglobin Genotype–Stratified Diabetes: A Systematic Review of Randomized Controlled Trials

**DOI:** 10.1155/2024/6645595

**Published:** 2024-10-21

**Authors:** Pan-pan Zheng, Li-wen Zhang, Dan Sheng, Min-zhen Wang, Rong Li, Wei-li Zhao, Rongmei Liu, Xian Xiu, Yu-sha Zhao, Xi Min, Zhi-kai Wang, Zan-chao Liu

**Affiliations:** ^1^Hebei Key Laboratory of Basic Medicine for Diabetes, The Second Hospital of Shijiazhuang 050000, Shijiazhuang, Hebei, China; ^2^Hebei Key Laboratory of Environment and Human Health, Department of Epidemiology and Statistics, School of Public Health, Hebei Medical University 050017, Shijiazhuang, Hebei, China; ^3^Institute of Epidemiology and Health Statistics, School of Public Health, Lanzhou University 730030, Lanzhou, Gansu, China; ^4^Shijiazhuang Technology Innovation Center of Precision Medicine for Diabetes, The Second Hospital of Shijiazhuang 050000, Shijiazhuang, Hebei, China; ^5^Department of Clinical Laboratories, Gaoxin District Changjiang Community Health Center 050000, Shijiazhuang, Hebei, China; ^6^School of Clinical Medicine, Hebei Medical University 050017, Shijiazhuang, Hebei, China

## Abstract

**Background:** Vitamin E, an essential micronutrient with antioxidant potential, can dramatically reduce the cardiovascular risk in individuals with haptoglobin (Hp) 2-2 genotype diabetes; however, the underlying mechanism remains unclear.

**Objective:** The objective of this study is to evaluate the effect of vitamin E supplementation on high-density lipoprotein (HDL) levels and function in individuals with diabetes stratified by Hp genotype.

**Methods:** All relevant studies published up to May 2023 were systematically reviewed using PubMed, Cochrane Library, Web of Science, Chinese Wanfang, China Science and Technology Journal, and Chinese National Knowledge Infrastructure databases. Randomized controlled trials that evaluated the effects of vitamin E supplementation on HDL levels were included. The outcomes assessed were changes in HDL concentrations, cholesterol efflux, and HDL-associated lipid peroxides.

**Results:** In total, 163 publications were selected. Based on inclusion and exclusion selection and quality assessment, five studies with 463 participants were included. Vitamin E supplementation did not exert any effect on HDL levels in individuals with diabetes with any Hp genotype. Three of the five studies revealed that vitamin E improved cholesterol efflux and HDL lipid peroxides in individuals with Hp2-2 diabetes but did not positively impact HDL function in Hp1 carriers.

**Conclusions:** Although vitamin E supplementation did not significantly impact HDL levels in individuals with diabetes of any Hp genotype, it may improve HDL function in individuals with Hp2-2 diabetes. These findings indicate a pharmacogenetic interaction between vitamin E and the Hp genotype on HDL function. Moreover, vitamin E supplementation may be an effective strategy for specific individuals with diabetes.

## 1. Introduction

Diabetes mellitus (DM), a noncommunicable chronic disease affecting 537 million adults, has gradually become one of the most crucial public health problems worldwide [1–3]. DM increases the risk of microvascular and macrovascular complications. Individuals with DM have a 2–4-fold increased risk of developing cardiovascular diseases (CVDs) [4]. CVDs are the leading cause of morbidity and mortality in patients with DM and can cause death in > 50% of patients with type 2 DM [5]. Oxidative stress and inflammation due to hyperglycemia are key factors in the development and complications of DM [6, 7].

Haptoglobin (Hp) is an acute response protein known to occur in both mammals and humans. Its major role is to capture free hemoglobin (Hb) released during hemolysis to form the Hp–Hb complex, which is cleared by the scavenger receptor CD163 on monocytes and macrophages [8, 9]. Hp exerts an important antioxidant and anti-inflammatory role by regulating the fate of Hb and its iron cargo [10, 11]. Genetic polymorphisms in human Hp lead to two dominant alleles, Hp1 and Hp2, on chromosome 16q22, forming three genotypes: Hp1-1, Hp2-2, and Hp2-1 [12]. Hp1-1 has the highest antioxidant and anti-inflammatory capacity, while Hp2-2 has the lowest activity, with Hp2-1 displaying intermediate activity [8, 9]. A meta-analysis has also confirmed that the Hp2-2 genotype is positively associated with a high risk of CVD in patients with diabetes [5] owing to the impaired antioxidant function of the Hp2-2 protein [13].

Vitamin E (alpha-tocopherol) is a potent antioxidant owing to its ability to scavenge free radicals and singlet oxygen [14]. Vitamin E treatment is clinically beneficial for glycemic control and can alleviate oxidative stress-induced pancreatic *β*-cell dysfunction [14, 15]. The role of vitamin E in preventing diabetes-related CVD is reportedly Hp genotype–dependent [16]. Clinical studies have shown that vitamin E can reduce CVD events in individuals with Hp2-2 phenotype diabetes [17]. According to a meta-analysis, vitamin E administration remarkably decreased CV mortality in the Hp2-2 DM cohort when compared with that in the non-Hp2-2 DM cohort [16].

High-density lipoprotein (HDL) is considered an antiatherogenic lipoprotein, and the consensus has shifted toward HDL as a key protective factor against CVD [18]. Reportedly, the protective anti-inflammatory properties of HDL and its ability to reverse cholesterol transport may be lost in the presence of DM, possibly due to oxidative modification of its major constituent protein, apolipoprotein A-I (ApoA1) [16, 19, 20]. Hp is an HDL-related protein that binds to ApoA1 [21]. In patients with Hp2-2 diabetes, Hp interacts with ApoA1, enhancing the quantity of HDL associated with Hp–Hb [16]. Serum HDL–mediated cholesterol efflux was found to differ substantially among patients with different Hp phenotype diabetes [20]. A clinical study has shown that vitamin E concentration is positively correlated with HDL [22] and that vitamin E can improve HDL function in patients with end-stage kidney disease [23]. Therefore, vitamin E may be a readily available therapeutic option for patients with increased oxidative stress, such as those with DM with the Hp2-2 phenotype, which may be partially achieved by improving HDL levels.

The effect of vitamin E on HDL function has been extensively explored in patients with DM with different Hp phenotypes; however, most studies involved small sample sizes, single-center designs, and inconsistent conclusions. In this literature review, we aimed to summarize the current evidence from all published randomized controlled studies evaluating the influence of vitamin E management on HDL levels in patients with Hp genotype–stratified DM and to identify gaps in these studies.

## 2. Methods

This systematic review was conducted in accordance with the Preferred Reporting Items for Systematic Reviews and Meta-Analyses guidelines [24]. The protocol was registered in PROSPERO (CRD42023442947). ROB v1 was used to assess the risk of bias.

### 2.1. Database and Search Strategies

To conduct this systematic review, relevant studies published in English or Chinese were retrieved from the electronic databases of PubMed, Cochrane Library, Web of Science, Chinese Wanfang, China Science and Technology Journal, and Chinese National Knowledge Infrastructure (CNKI) from their inception to May 2023. We used the following search terms: (“vitamin E” OR “tocopherol”) AND (“diabetes mellitus” OR “diabetes” OR “mellitus”) AND (“haptoglobin” OR “haptoglobin genotype” OR “Hp”), and the details of the search are presented in Table S1. In addition, reference lists from the initial database search were manually screened to identify additional potential studies.

### 2.2. Inclusion and Exclusion Criteria

All related human randomized controlled trials (RCTs) were included based on the following inclusion criteria: (i) participants diagnosed with type 1 or type 2 diabetes, (ii) RCTs with placebo, (iii) RCTs with durations ≥ 8 weeks, (iv) RCTs of Hp genotyping used to compare outcomes between patients with and without the Hp2-2 genotype, and (v) reported at least one of the three outcomes of interest (HDL concentrations, cholesterol efflux, and HDL lipid peroxides). Exclusion criteria were as follows: (i) review/meta-analysis/editorial/others, (ii) no intervention/nonrelevant/irrelevant original data, and (iii) animal studies/animal-relevant outcome/relevant outcomes, including patients without diabetes from the same studies.

### 2.3. Data Extraction and Bias Risk Assessment

Two authors (P.Z. and D.S.) independently extracted data from the five eligible trials using Microsoft Excel. The following details of the included trials were extracted: study information, participant characteristics, vitamin E supplements (type and dosage), route, control group, sample size, study duration, and selective outcomes. All information was cross-checked twice. After the initial assessment, the two authors independently assessed the eligibility of the RCTs identified for potential inclusion using Review Manager version 5.4. Disagreements between the two authors on the subject matter or risk of bias assessment were deliberated with a third author (M.Z.), who reviewed all extracted data to check the accuracy of the information from the original articles.

### 2.4. Data Analysis

Owing to the heterogeneity of the primary outcome measures (comprehension), inconsistencies in statistical formats, and the absence of original data, we were unable to conduct a meta-analysis. Therefore, we have classified and listed the primary outcomes of all eligible trials with accurate *p* values for intergroup comparisons using appropriate footnotes to help readers understand the comments.

## 3. Results

### 3.1. Search Results

**Figure 1:**
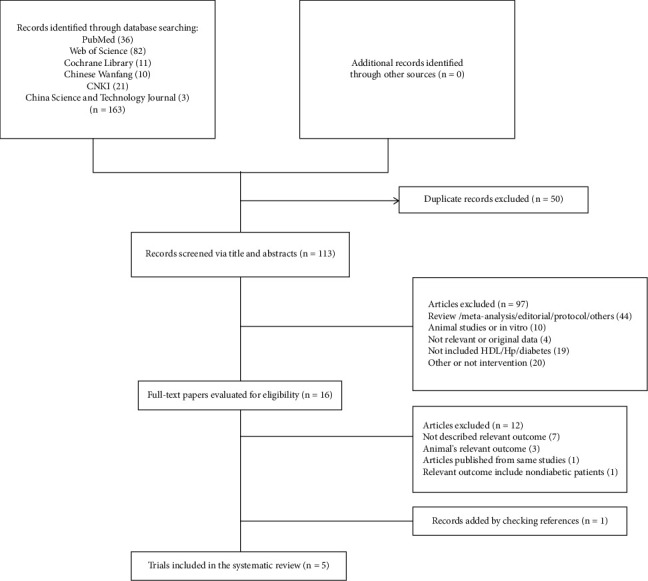
Flow diagram of the study screening process.

Figure 1 presents a flow chart of the literature search results and the study selection process. Initially, we identified 163 relevant studies. Of these, 50 were excluded due to duplication, and 97 were irrelevant after screening the titles and abstracts. Subsequently, 12 studies were excluded after reviewing the entire report, as they lacked relevant results or included the wrong type of results (*n* = 10), the wrong type of population (*n* = 1), or originated from the same study (*n* = 1), with Table S2 presenting additional details. One trial was manually identified from the list of references of included studies. Finally, five RCTs were included in the current systematic review [25–29].

### 3.2. Study Characteristics

All five RCTs were published between 2004 and 2020 and included 443 patients with DM from different countries. Vitamin E was mainly supplemented at a daily dose of 400 IU, whereas the control group was administered a placebo. The duration of intervention ranged from 8 weeks to 2.8 ± 0.9 years.  Table 1 summarizes the general characteristics of all included studies. The included studies involved patients from the United States (five clinical centers), Canada (two clinical centers) [25], Israel [26, 27], United States (two type 1 diabetes registries) [28], and Singapore [29]. Among these studies, three were multicenter [25, 26, 28], and three had crossover design [26–28]. Considering the diabetes types, one study involved patients with type 1 [28], two comprised patients with type 2 [26, 29], and two were not provided [25, 27]. One study included only females [25], two included both males and females [28, 29], and two failed to report the sex composition [26, 27]. The daily dose of vitamin E supplementation was 400 IU in four studies [26–29] and 800 IU in one study [25]. Two studies analyzed HDL concentrations [25, 29], one determined lipid peroxides and cholesterol efflux [26], and two determined HDL concentrations, lipid peroxides, and cholesterol efflux [27, 28]. The outcomes were presented as changes before and after treatment [25], *p* values compared with baseline [26], mean ± standard error of the mean (SEM) [27], *β* (standard error) [28], mean (interquartile range [IQR]) [29], respectively.

### 3.3. Risk of Bias Assessment

**Figure 2:**
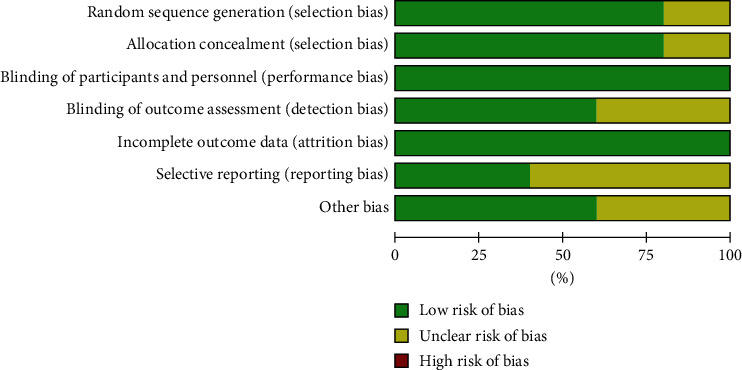
Risk of bias of all included studies.

**Figure 3:**
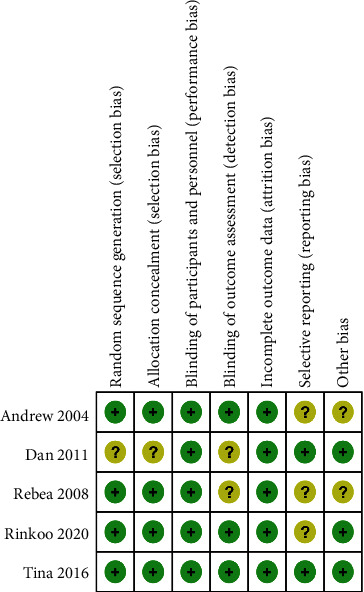
Risk of bias summary for each included study.

As shown in Figures 2 and 3, we assessed the quality and the risk of bias of the included RCTs. We classified the risk of bias as “low,” “unclear,” or “high.” If the information was insufficient to exclude any relevant risks, the domains were considered as having an unclear risk of bias.

As shown in the figures, four RCTs used randomization sequence generation methods, such as a 2 × 2 factorial design [25], computerized randomization sequence [26], or blocked randomization schedule [28, 29]. One study was judged to have an unclear risk of bias due to allocation concealment; therefore, we judged this as unclear [27]. All five included RCTs clearly reported the blinding of participants and personnel. Three trials explicitly mentioned that the researchers were unaware of the results [25, 28, 29]. All five included studies reported complete data for each primary outcome indicator. Three trials had unclear risks of bias due to selective reporting [25, 26, 29]. In addition, three trials were scored as having other biases because conflicts of interest were not considered [25, 28, 29]. Overall, the included studies had good quality, including three good-quality (two or less “unclear”) studies and two moderate-quality (one “unclear” or one “high”) studies. More than three “unclear” or two or more “high” are considered low-quality studies.

### 3.4. The Impact of Vitamin E Supplementation on HDL Concentration Level

Four trials [25, 27–29] compared HDL concentrations in patients with non-Hp2-2 and Hp2-2 genotype DM between the vitamin E and placebo groups. Of these, two trials [25, 28] simultaneously compared HDL concentrations of three Hp genotypes, one trial [27] compared HDL concentrations with Hp2-1 and Hp2-2 genotypes, and one [29] identified Hp1 carriers as the non-Hp2-2 group, with the results tabulated in the Hp1-1 table for visual presentation. Vitamin E did not exert any effect on HDL levels in patients with Hp1-1 (*p* = 0.39, 0.85, 0.478), Hp2-1 (*p* = 0.13, 0.75, 0.81), and Hp2-2 (*p* = 0.08, 0.88, 0.30, 0.972) when compared with the placebo (Table 2).

### 3.5. The Impact of Vitamin E Supplementation on Cholesterol Efflux

Three trials [26–28] compared cholesterol efflux in DM patients with non-HP2-2 and Hp2-2 genotypes between the vitamin E and placebo groups. Of these, one trial [28] simultaneously compared cholesterol efflux with three Hp genotypes and found that supplementation with vitamin E exerted no effect on cholesterol efflux in Hp1 carriers (*p* = 0.72, 0.81) but increased cholesterol efflux in Hp2-2 carriers (*β* = 0.79, *p* = 0.03). One trial [27] compared cholesterol efflux in patients carrying Hp2-1 and Hp2-2 genotypes, revealing a small decrease in cholesterol efflux in Hp2-1 carriers (*p* = 0.04), as well as a small increase in cholesterol efflux in Hp2-2 carriers (*p* = 0.05). Another trial [26] examined cholesterol efflux among patients with Hp2-2 DM and showed that supplementation with vitamin E enhanced cholesterol efflux (*p* = 0.04) (Table 3).

### 3.6. The Impact of Vitamin E Supplementation on HDL-Associated Lipid Peroxides

Of the five RCTs, three compared the effects of vitamin E and placebo on HDL-associated lipid peroxides in patients with non-Hp2-2 and Hp2-2 DM [26–28] (*n* = 164 patients). One trial [28] showed that supplementation with vitamin E could increase lipid peroxides in the Hp1-1 cohort (*β* = 0.18, *p* = 0.05) but had no significant effect on Hp2 carriers (*p* = 0.07, 0.60). One trial [27] found that supplementation with vitamin E reduced lipid peroxide levels by nearly 50% in Hp2-2 cells (*p* = 0.003) but did not affect Hp2-1 cells (*p* = 0.95). One trial [26] demonstrated that vitamin E supplementation could suppress levels of lipid peroxides in patients with Hp2-2 DM when compared with the placebo (*p* = 0.01) (Table 4).

## 4. Discussion

DM is a growing global burden, and there is a pressing need to identify and develop appropriate prevention, diagnosis, and therapeutic strategies [30]. As an incurable and heterogeneous disease, DM manifestations and complications vary interindividually [31]. There is widespread evidence supporting the notion that therapy needs to migrate to a platform for precise clinical management of patients based on individual genetic information [32]. Genetic polymorphisms can influence an individual's response to certain drugs, and polymorphisms in certain genes have been found to affect an individual's response to antidiabetic drugs [33, 34]. Therefore, a better understanding of the relationship between genetic polymorphisms and drug therapy could facilitate the development of individualized treatments for diabetes. Although the association between the antioxidant vitamin E and diabetic CVD events has been well documented [16], the underlying molecular mechanisms remain unclear. Identifying these molecular linkage pathways will assist in launching a new branch of individualized and accurate diagnosis and treatment for DM. In this systematic review, the results of five RCTs, comprising 443 patients, revealed that vitamin E supplementation may substantially improve cholesterol efflux and reduce lipid peroxide levels in the Hp2-2 subgroup, with no significant impact on the serum HDL levels of all Hp genotypes, revealing the potential advantage of selective vitamin E use in improving HDL function in patients with Hp2-2 DM.

In the current systematic review, none of the five included studies detected any benefit of vitamin E supplementation on HDL-mediated lipid peroxides and cholesterol efflux in patients with Hp2-1 DM; however, one study found that supplementation with vitamin E could reduce cholesterol efflux in the Hp2-1 subgroup when compared with placebo treatment. Therefore, vitamin E (alpha-tocopherol) exerts no protective effect on HDL function in patients carrying Hp2-1 carriers. In addition, the data on the impact of vitamin E therapy on HDL function in Hp1-1 cells are limited. Only one study reported that vitamin E did not affect cholesterol efflux in Hp1-1 carriers, while another showed that vitamin E could increase lipid peroxide levels in Hp1-1 carriers. Accordingly, vitamin E did not protect HDL function in Hp1-1 carriers. Based on these results, vitamin E administration did not exert any positive effect on HDL function in Hp2-1 and Hp1-1 carriers. This result is consistent with the functional dependence of HDL on the Hp genotype [35].

The Hp2-2 genotype reportedly increases the risk of CVD in patients with DM. This can be attributed to the abnormal Hp protein structure of patients with Hp2-2 DM, leading to a delay in Hp–Hb complex clearance [26, 36]. The binding of the Hp2-2 protein to ApoA1 on HDL has been shown to promote the oxidative modification of HDL in patients with Hp2-2 DM, which leads to the inhibition of cholesterol efflux [26]. HDL-mediated dysfunction of cholesterol efflux may contribute to the increased susceptibility to CVD observed in the Hp2-2 genotype and type 1 DM [27]. This evidence supports the previously proposed explanation that the CVD risk associated with Hp2-2 is partly attributed to HDL dysfunction caused by the reduced antioxidant capacity of the Hp2 polymer [16]. Increased oxidative stress in the population with diabetes and the Hp2-2 genotype indicates that antioxidants may be potential therapeutics for reducing CVD risk in this group.

It should be noted that not all antioxidants are equivalent when evaluating the potential of antioxidant therapy against the CVD risk in patients with diabetes, and the benefits among specific patient subgroups cannot be ruled out [37]. Vitamin E has been shown to reduce the incidence of vascular events in individuals with defective antioxidant-defense genes [38]. In an experimental animal study, treatment with vitamin E completely blocked glycosylated Hb-Hp2-2–mediated oxidation and fully restored HDL function in mice with DM and Hp2-2 [39]. Among patients with type 2 DM or postmenopausal females with diabetes, there were no differences in altered HDL levels between any of the Hp subgroups [25, 29]. Another vitamin E intervention study in patients with diabetes revealed that HDL function was markedly elevated in Hp2-2 carriers but substantially reduced in Hp2-1 carriers [27]. In a study assessing patients with type 1 DM, vitamin E was shown to promote HDL function in Hp2-2 carriers but was detrimental to lipid peroxides in Hp1 carriers [28]. In patients with type 2 DM, vitamin E reportedly enhanced HDL function among Hp2-2 carriers but had no effect in patients with the Hp2-1 genotype [26]. Although HDL function appears to be Hp phenotype–dependent, a study on patients with end-stage renal failure undergoing hemodialysis, including 20 patients with diabetes and 20 nondiabetic patients, revealed that vitamin E supplementation improved HDL function independent of diabetes and Hp phenotypes [23]. In the current systematic review, we found that vitamin E could improve HDL function in patients with Hp2-2 diabetes but not in Hp1 carriers.

Our systematic review suggests that drug-genetic interactions between vitamin E and the Hp genotype may be achieved partly by improving the biological function of HDL in DM, which supports the hypothesis that individuals with a high CVD risk carrying the Hp2-2 genotype may benefit from vitamin E therapy. In clinical settings, physicians should consider detecting the Hp genotype of patients with DM and provide an appropriate dose of vitamin E to patients carrying the Hp2-2 genotype, which may help restore HDL function, thus reducing the occurrence of CVD. Given the limited number of studies included in our systematic review and inconsistent data forms, a meta-analysis could not be performed. A few included studies lacked certain data, such as sex and age; therefore, it is unclear whether sex and age impacted the final results. The small sample size and heterogeneity of the results indicate that these results need to be replicated in large-scale multicenter prospective studies that include maximum follow-up periods and report detailed and specific cardiovascular outcomes in a uniform data format. Specialized animal or cellular experiments are also needed to explore pharmacogenetic interactions and possible molecular mechanisms, as well as to address any remaining uncertainties.

## 5. Conclusions

To the best of our knowledge, this is the first systematic review of RCTs evaluating the effects of vitamin E (alpha-tocopherol) supplementation on HDL levels in patients with Hp genotype–stratified DM. Our findings revealed that vitamin E could exert a positive and substantial clinical effect on HDL function in individuals with DM and Hp2-2 but no positive effect in Hp1 carriers, thereby suggesting that the impact of vitamin E therapy on HDL function in DM is Hp genotype–dependent.

## Figures and Tables

**Table 1:** Characteristic of included studies in systematic review.

**Author, year**	**Country**	**Design**	**Type of diabetes**	**Sex (M/F)**	**Mean age**	**Vitamin E dose**	**Route**	**Control group**	**Sample size**	**Duration**	**Selective outcomes (data form)**
Andrew, 2004	United States and Canada	RP/PC/DB	Diabetes	F	NA	Vitamin E, 400 IU	Twice daily	Placebo	113	2.8 ± 0.9 years	HDL concentrations (change)
Rabea, 2008	Israel	RC/PC/DB	Type 2 diabetes	NA	60 ± 7	*d*-Tocopherol, 400 IU	Daily	Placebo	18	2 months	Cholesterol efflux (*P*), HDL lipid peroxides (*P*)
Dan, 2011	Israel	RC/PC/DB	Diabetes	NA	NA	*d*-Alpha-tocopheryl acetate, 400 IU	Daily	Placebo	59	3 months	Cholesterol efflux (mean ± SEM), HDL lipid peroxides (mean ± SEM), HDL concentrations (mean ± SEM)
Tina, 2016	United States	RC/PC/DB	Type 1 diabetes	M/F	NA	*α*-Tocopherol, 400 IU	Daily	Placebo	87	8 weeks	Cholesterol efflux (*β*[std. error]), HDL lipid peroxides (*β*[std. error]), HDL concentrations (*β*[std. error])
Rinkoo, 2020	Singapore	R/PC/DB	Type 2 diabetes	M/F	56 ± 10	Alpha-tocopherol, 400 IU	Daily	Placebo	166	24 weeks	HDL concentrations (mean [IQR])

Abbreviations: F: female; HDL: high-density lipoprotein; M: male; NA: not available; R/PC/DB: randomized, double-blind, placebo-controlled trial; RC/PC/DB: randomized, cross-over, double-blind, placebo-controlled trial; RP/PC/DB: randomized, prospective, double-blind, placebo-controlled trial.

**Table 2:** Pooled estimates of the effect of vitamin E on HDL levels in DM patients with different Hp genotypes.

**Authors, year**	**Hp1-1**				**Hp2-1**				**Hp2-2**			
	**n**	**Placebo**	**Vitamin E**	**p**	**n**	**Placebo**	**Vitamin E**	**p**	**n**	**Placebo**	**Vitamin E**	**p**
Andrew, 2004	23	−0.67^a^	5.15^a^	0.39	58	0.38^a^	4.19^a^	0.13	32	−0.6^a^	7.8^a^	0.08
Dan, 2011	—	—	—	—	31	46.903 ± 2.229^b^	47.613 ± 1.916^b^	0.81	28	42.86 ± 1.59^b^	43.75 ± 1.85^b^	0.30
Tina, 2016	27	0.20 (1.03)^c^		0.85	31	0.32 (1.02)^c^		0.75	29	−0.20 (1.25)^c^		0.88
Rinkoo, 2020	80	1.10 (0.30)^d^	1.10 (0.40)^d^	0.478	—	—	—	—	86	1.10 (0.28)^d^	1.10 (0.23)^d^	0.972

*Note:* “—”: unknown.

^a^Change.

^b^Mean ± SEM (mg/dL).

^c^ *β*(std. error).

^d^Mean (IQR) (mmol/L).

**Table 3:** Pooled estimates of the effect of vitamin E on cholesterol efflux in DM patients with different Hp genotype.

**Authors, year**	**Hp1-1**				**Hp2-1**				**Hp2-2**			
	**n**	**Placebo**	**Vitamin E**	**p**	**n**	**Placebo**	**Vitamin E**	**p**	**n**	**Placebo**	**Vitamin E**	**p**
Rabea, 2008	—	—	—	—	—	—	—	—	18	0.33^a^	0.004^a^	0.04
Dan, 2011	—	—	—	—	31	15.40 ± 1.00^b^	13.90 ± 1.09^b^	0.04	28	11.10 ± 0.64^b^	12.10 ± 0.81^b^	0.05
Tina, 2016	27	0.13 (0.36)^c^		0.72	31	0.08 (0.33)^c^		0.81	29	0.79 (0.36)^c^		0.03

*Note:* “—”: unknown.

^a^ *p*: compared to baseline.

^b^Mean ± SEM (%).

^c^ *β*(std. error).

**Table 4:** Pooled estimates of the effect of vitamin E on HDL-associated lipid peroxides in DM patients with different Hp genotypes.

**Authors, year**	**Hp1-1**				**Hp2-1**				**Hp2-2**			
	**n**	**Placebo**	**Vitamin E**	**p**	**n**	**Placebo**	**Vitamin E**	**p**	**n**	**Placebo**	**Vitamin E**	**p**
Rabea, 2008	—	—	—	—	—	—	—	—	18	0.35^a^	0.03^a^	0.01
Dan, 2011	—	—	—	—	31	1.071 ± 0.174^b^	1.065 ± 0.156^b^	0.95	28	1.070 ± 0.190^b^	0.549 ± 0.104^b^	0.003
Tina, 2016	27	0.18 (0.09)^c^		0.05	31	0.21 (0.11)^c^		0.07	29	0.07 (0.12)^c^		0.60

*Note:* “—”: unknown.

^a^ *p*: compared to baseline.

^b^Mean ± SEM (nmol/*μ*g).

^c^ *β*(std. error).

## Data Availability

The datasets generated during and/or analyzed during the current study are available from the corresponding author on a reasonable request.

## Supporting Information

Supporting Information 1 Table S1: the details of the database and search strategies.

Supporting Information 2 Table S2: excluded studies and their reasons after reading the entire literature.
