# Supplementary material for: Impact of Vitamin E Supplementation on High-Density Lipoprotein in Patients With Haptoglobin Genotype–Stratified Diabetes: A Systematic Review of Randomized Controlled Trials
Source: J Diabetes Res. 2024 Oct 21;2024:6645595. doi: 10.1155/2024/6645595 (PMC11519069; doi:10.1155/2024/6645595)
Supplement: Supporting Information 1 — Table S1: the details of the database and search strategies. [file 6645595.f1.docx]

# Table S1. Search strategies.

# Search strategy in PubMed

#1 "Vitamin E"[MeSH Terms] OR "Vitamin"[Title/Abstract] OR "alpha-tocopherol"[MeSH Terms] OR "tocopherol"[Title/Abstract] OR "Vitamin E"[Title/Abstract]

#2 "Diabetes Mellitus"[MeSH Terms] OR "diabetes mellitus, type 2"[MeSH Terms] OR "diabetes"[Title/Abstract] OR "mellitus"[Title/Abstract] OR "Diabetes Mellitus"[Title/Abstract]

#3 "haptoglobins"[MeSH Terms] OR "haptoglobins"[All Fields] OR "haptoglobin"[All Fields] AND "genotype"[MeSH Terms] OR "haptoglobin 2 2 genotype"[Title/Abstract] OR "haptoglobin genotype"[Title/Abstract] OR "Hp2-2"[Title/Abstract]

#4 #1 AND #2 AND #3

# Cochrane Library

# #1 (vitamin E):ti,ab,kw OR (alpha-tocopherol):ti,ab,kw

# #2 (diabetes mellitus):ti,ab,kw OR (diabetes):ti,ab,kw OR (mellitus):ti,ab,kw

# #3 (haptoglobin 22 genotype):ti,ab,kw OR (haptoglobin genotype):ti,ab,kw

# #4 #1 AND #2 AND #3

**Web of Science**

# (((vitamin E) OR (alpha-tocopherol)) AND (((diabetes mellitus) OR (diabetes)) OR (mellitus))) AND ((haptoglobin 2-2 genotype) OR (haptoglobin genotype)))
