# Supplementary material for: Impact of Vitamin E Supplementation on High-Density Lipoprotein in Patients With Haptoglobin Genotype–Stratified Diabetes: A Systematic Review of Randomized Controlled Trials
Source: J Diabetes Res. 2024 Oct 21;2024:6645595. doi: 10.1155/2024/6645595 (PMC11519069; doi:10.1155/2024/6645595)
Supplement: Supporting Information 2 — Table S2: excluded studies and their reasons after reading the entire literature. [file 6645595.f2.docx]

| Title of the original literature | DOI/Journal, Volume (Issue) | Excluded reason |
| --- | --- | --- |
| Precision Healthcare of Type 2 Diabetic Patients Through Implementation of HaptoglobinGenotyping | 10.3389/fcvm.2018.00141 | Not described relevant outcome |
| Haptoglobin Genotype and Renal Function Decline in Type 1 Diabetes | 10.2337/db09-0874 | Not described relevant outcome |
| Haptoglobin Genotype Is a Determinant of Hemoglobin Adducts and Vitamin E Content in HDL | 10.1155/2018/6125420 | Not described relevant outcome |
| Vitamin E Intake Is Associated with Lower Brain Volume in Haptoglobin 1-1 Elderly with Type 2 Diabetes | 10.3233/JAD-191294 | Not described relevant outcome |
| Haptoglobin phenotype, pre-eclampsia, and response to supplementation with vitamins C and E in pregnant women with type-1 diabetes | 10.1111/1471-0528.12288 | Not described relevant outcome |
| Anti-oxidative Treatment with Vitamin E Improves Peripheral Vascular Function in Patients with Diabetes Mellitus and Haptoglobin 2-2 Genotype: A Double-blinded Cross-over Study | 10.1016/j.diabres.2017.06.026 | Not described relevant outcome |
| Vitamin E Supplementation Reduces Cardiovascular Events in a Subgroup of Middle-Aged Individuals With Both Type 2 Diabetes Mellitus and the Haptoglobin 2-2 Genotype A Prospective Double-Blinded Clinical Trial | 10.1161/ATVBAHA.107.153965 | Not described relevant outcome |
| Divergent Effects of a-Tocopherol and Vitamin C on the Generation of Dysfunctional HDL Associated with Diabetes and the Hp 2-2 Genotype | 10.1089/ars.2009.2829 | Animal’s relevant outcome |
| Targeting HDL Quality Rather than Quantity: Providing the Mechanistic Rationale for the Pharmacogenomic Interaction between the Haptoglobin Genotype and Vitamin E on Cardiovascular Disease In Individuals with Diabetes Mellitus | Circulation, 118(18) | Animal’s relevant outcome |
| Correction of HDL dysfunction in individuals with diabetes and the haptoglobin 2-2 genotype | 10.2337/db08-0450 | Animal’s relevant outcome |
| Vitamin E Supplementation is Associated With Increased Cardiovascular Disease and Mortality in Individuals With the Hp 2-1 Genotype and Diabetes | Circulation, 122(21) | Articles published from same studies |
| Vitamin E supplementation improves highdensitiy lipoprotein and endothelial functions in end-stage kidney disease patients undergoing hemodialysis | 10.5414/CN109197 | Relevant outcome include non-diabetic patients |

Table S2 Excluded studies and their reasons after reading the entire literature.
